# Extraocular Sebaceous Carcinoma in Lynch Syndrome: A Sentinel Cutaneous Clue to Muir-Torre Syndrome

**DOI:** 10.7759/cureus.88267

**Published:** 2025-07-18

**Authors:** Suyog S Dhamale, Anshu Baghel, Kusumika Kanak, Vidyadhar Sardesai

**Affiliations:** 1 Department of Dermatology, Venereology and Leprosy, Bharati Vidyapeeth Medical College, Pune, IND; 2 Department of Dermatology, King Edward Memorial Hospital, Pune, IND

**Keywords:** cutaneous malignancy, extraocular, lynch syndrome, mohs micrographic surgery, muir-torre syndrome, sebaceous carcinoma

## Abstract

Sebaceous carcinoma (SC) is a rare and aggressive cutaneous malignancy, most commonly arising in the periocular region. Extraocular presentations, particularly in patients with Lynch syndrome (LS), are uncommon but clinically significant. We report the case of a 70-year-old male with a known diagnosis of LS and a strong family history of visceral malignancies, who presented with a rapidly enlarging lesion on the forehead. Histopathological examination confirmed extraocular sebaceous carcinoma with a high Ki-67 proliferative index. The lesion was managed successfully with Mohs micrographic surgery (MMS) to ensure complete excision and minimize recurrence. This case fulfilled clinical and molecular criteria for Muir-Torre syndrome (MTS), a phenotypic variant of LS characterized by sebaceous neoplasms and internal malignancies. It underscores the importance of recognizing cutaneous markers of hereditary cancer syndromes and emphasizes the need for prompt dermatological and genetic evaluation in at-risk individuals. Early intervention facilitates timely treatment and familial cancer surveillance.

## Introduction

Sebaceous carcinoma (SC) is a rare and aggressive cutaneous malignancy that arises from sebaceous glands. It typically affects older adults and most commonly involves the periocular region [1]. Due to its variable appearance, SC is frequently misdiagnosed as other skin conditions such as sebaceous hyperplasia, sebaceoma, squamous cell carcinoma, and basal cell carcinoma, leading to delays in diagnosis and treatment. Of note, SC can be a cutaneous manifestation of Muir-Torre syndrome (MTS), a variant of Lynch syndrome (LS) [2]. LS, also known as hereditary non-polyposis colorectal cancer (HNPCC), is an autosomal dominant disorder caused by germline mutations in DNA mismatch repair (MMR) genes, predisposing individuals to multiple malignancies, particularly colorectal and endometrial cancers [3]. When any suspicious cutaneous growth occurs in the setting of LS, they should prompt evaluation to rule out malignancy to prevent morbidity.

This case report describes a rare presentation of extraocular SC in a 70-year-old male with a clinical diagnosis of LS and a strong family history of multiple malignancies. It also underscores the need for increased clinical awareness of cutaneous signs of hereditary cancer syndromes and the importance of early evaluation in at-risk individuals.

## Case presentation

A 70-year-old male presented to the dermatology outpatient department with a complaint of a skin lesion on his forehead, located near the medial aspect of the right eyebrow. The lesion had developed three weeks prior and showed rapid growth, reaching approximately 1 cm in diameter. It was non-pruritic, non-tender, but prone to bleeding upon touch. On further history, it was revealed that the patient was a known case of LS. His family history was significant for multiple malignancies, predominantly colorectal cancer, affecting eight family members across three generations. Due to this strong familial predisposition, the patient had been undergoing annual colonoscopic surveillance, leading to the incidental diagnosis of colon carcinoma a few years back. Over the past decade, the patient had undergone multiple colonoscopies and surgical interventions for colonic adenocarcinoma, colonic adenomas, and urothelial carcinoma.

**Figure 1:**
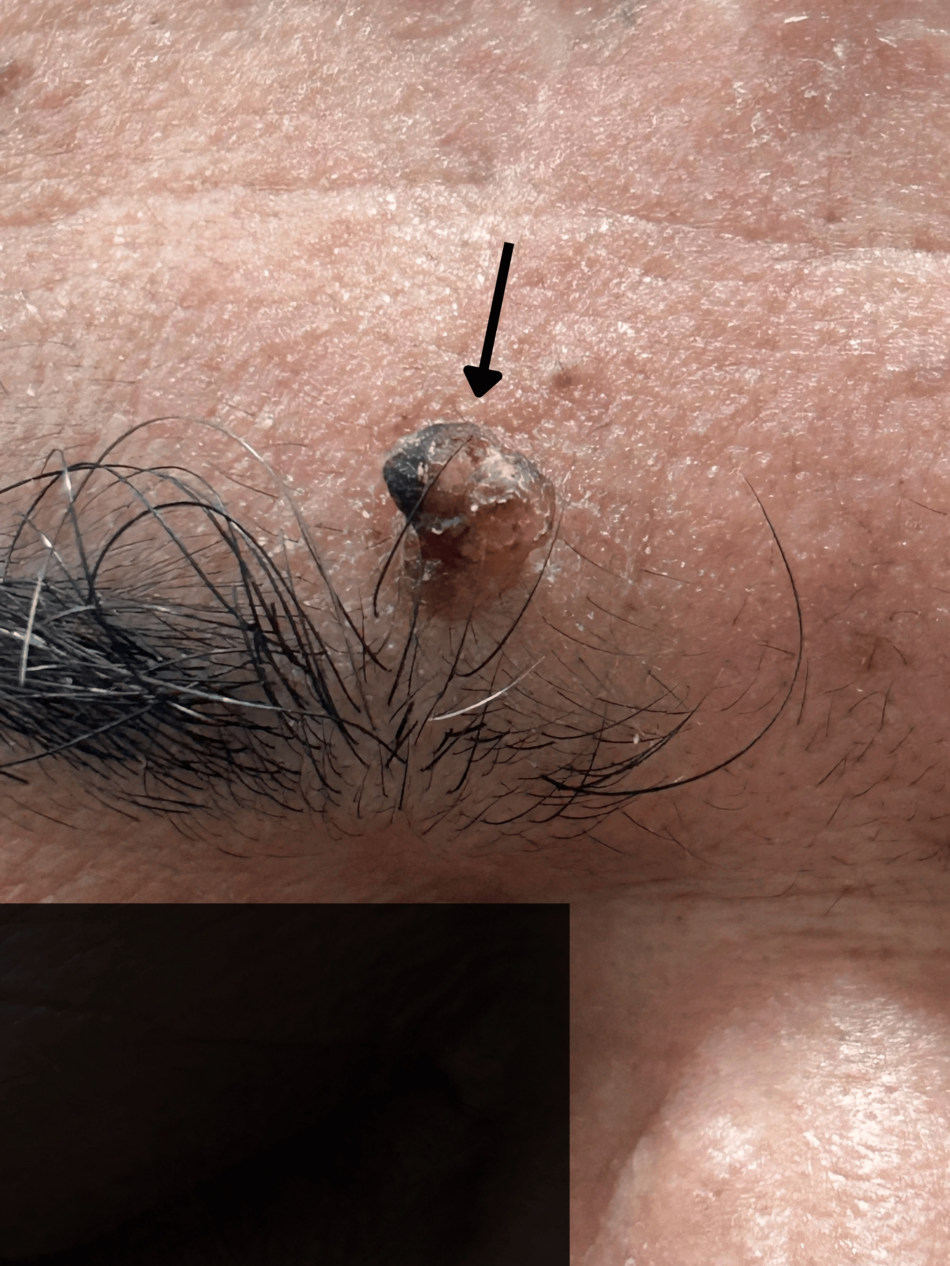
A solitary, pedunculated, hyperkeratotic mass (black arrow) over the superior aspect of medial end of right eyebrow

Although germline genetic testing, such as sequencing, was not performed due to financial and logistical limitations, a notable history of malignancies was present among first-degree relatives. Immunohistochemical analysis conducted on a tumor sample from a first-degree relative of the patient demonstrated mismatch repair protein deficiency (dMMR), with complete loss of nuclear expression of MSH1, MSH2, MSH6, and PMS2, findings consistent with LS. The patient had no other known comorbidities or drug allergies. His baseline laboratory investigations, including complete blood count, liver function tests, and renal function tests, were all within normal limits; hence, no significant abnormalities were noted. Dermatological examination revealed a solitary, pedunculated, hyperkeratotic mass measuring approximately 1 cm, located on the superior aspect of the medial right eyebrow (Figure 1).

**Figure 2:**
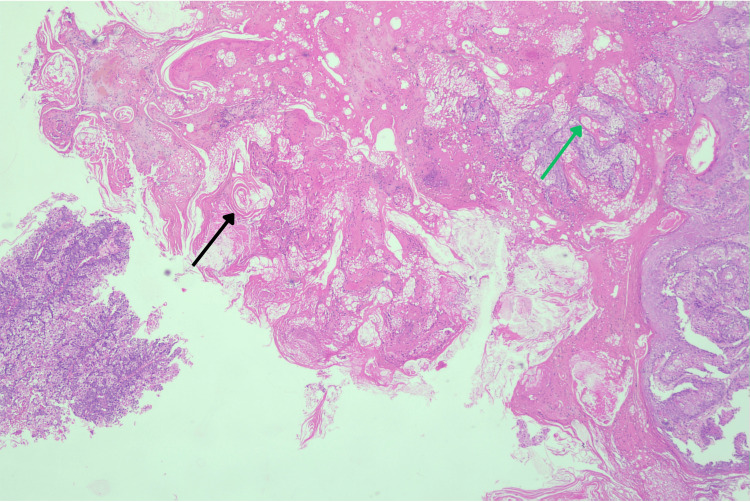
Histopathology (H&E, 10X) Scanner view depicting lobules of tumor cells with peripheral squamous differentiation (black arrow) and central tumor cells showing sebaceous morphology (green arrow)

The lesion was friable and bled upon palpation. Multiple seborrheic keratoses of varying sizes were also observed on the patient’s face. Given the rapid growth of the lesion, an excisional biopsy was performed for histopathological examination. Differential diagnoses considered included seborrheic keratosis, verruca (wart), pyogenic granuloma, and SC. Histopathological examination of the biopsy sample revealed multiple tumor lobules with characteristic peripheral squamous differentiation and central sebaceous morphology (Figure 2).

**Figure 3:**
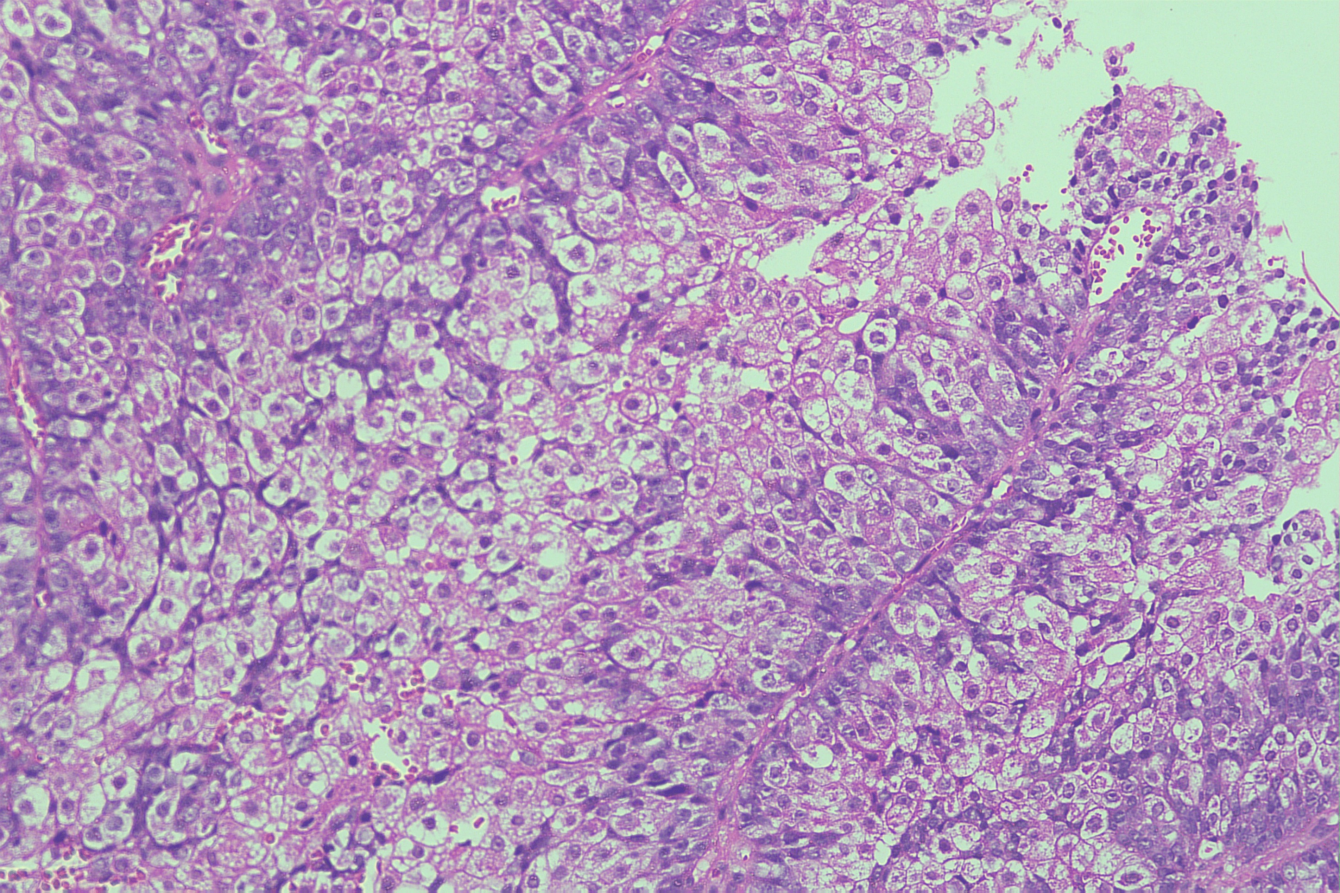
Histopathology (H&E, 40X) High-power view illustrating tumor cells with abundant eosinophilic to foamy cytoplasm, characteristic of sebaceous differentiation

Tumor cells exhibited marked pleomorphism with large polygonal shapes, vesicular nuclei, prominent eosinophilic nucleoli, and abundant eosinophilic to foamy cytoplasm (Figure 3).

**Figure 4:**
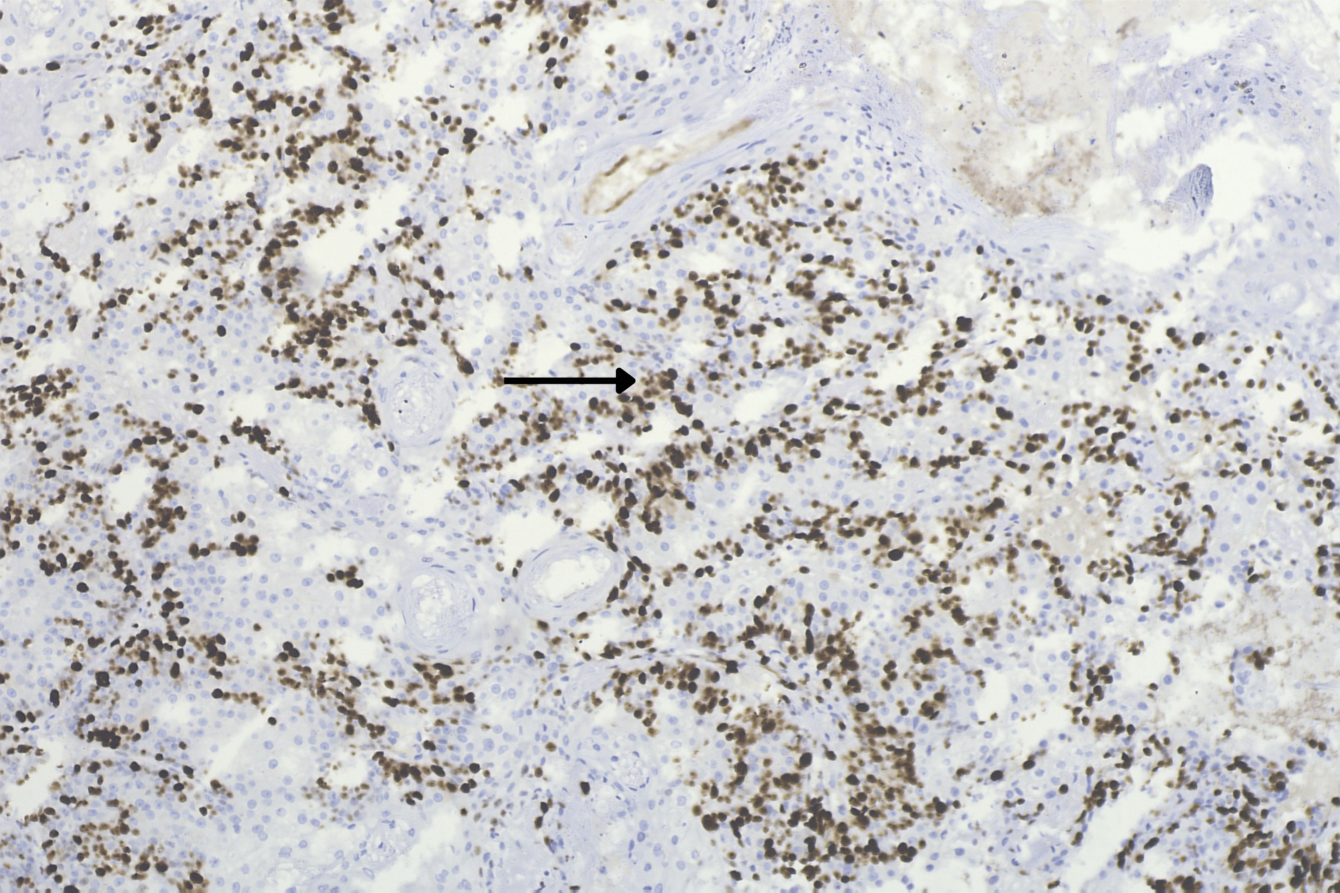
Immunohistochemistry for Ki-67 showing a high labeling index of 60% within malignant cells (black arrow)

Several atypical mitotic figures were observed, along with foci of necrosis surrounding the lobules. Additionally, one biopsy fragment showed hyperkeratosis and parakeratosis of the overlying epidermis, with acute inflammatory cell infiltration and bacterial colonies. Immunohistochemical marker Ki-67 was positive with a proliferative index of 60% (Figure 4).

Based on the history, clinical and histopathological findings, the diagnosis of extraocular SC associated with LS/MTS was ascertained. Given the location of the lesion, aggressive nature of SC, and its potential for local recurrence, Mohs micrographic surgery (MMS) was performed in this case to ensure complete excision of the SC with minimal removal of healthy tissue. After surgery, the patient was monitored for local recurrence. He has now completed four months of postoperative follow-up, during which no local recurrence or metastatic spread was observed. He continues to be under routine dermatologic surveillance. The patient was also evaluated by an oncologist, and no findings suggestive of regional extension or distant metastasis were observed.

## Discussion

SC is a rare and potentially aggressive malignancy arising from sebaceous glands [1]. It is broadly classified into two types: ocular and extraocular. Periorbital SC is the more common type and constitutes approximately 75% of cases. In contrast, extraocular SC is relatively uncommon, accounting for approximately 25% of all SC cases and often escapes early detection [2,4]. It may present in various anatomical locations, including the head and neck region (most commonly), scalp, trunk, upper extremities, and genital areas. Extraocular SC often presents with non-specific features and may mimic benign lesions like verruca, seborrheic keratosis, or pyogenic granuloma, leading to diagnostic delays and inappropriate treatment [1,2]. In our case, the lesion showed rapid progression within three weeks in a patient with LS with a history of prior visceral malignancies, prompting early biopsy and excision. Given its resemblance to basal or squamous cell carcinoma, histopathology remains essential for diagnosis. A high Ki-67 index in this case indicated significant proliferative activity, supporting the tumor’s aggressive nature and the need for prompt intervention.

An important aspect of this case is the patient’s underlying diagnosis of LS, a hereditary condition caused by inherited mutations in the DNA MMR genes. When sebaceous tumors develop in individuals with LS, the condition is known as MTS. MTS, a genodermatosis, is a rare skin-related form of LS, defined by the presence of at least one sebaceous tumor along with one visceral malignancy linked to MMR gene deficiency [2,3,5]. In our case, the patient’s history of colonic adenocarcinoma and urothelial carcinoma, combined with the recent development of SC and a strong family history of colorectal cancers across three generations, fulfils the clinical criteria. Moreover, the loss of mismatch repair protein (MSH1, MSH2, MSH6, PMS2) in the first-degree relative of the patient further substantiates the likely hereditary origin of MMR dysfunction, thus satisfying both the clinical and molecular dimensions of MTS [2]. Notably, extraocular SC is a rare but diagnostically significant finding in the context of LS and MTS. Although most SCs in MTS occur in the periocular region, extraocular presentations should not be overlooked, especially in high-risk individuals [2]. These tumors may serve as sentinel cutaneous markers of underlying hereditary cancer syndromes, and early identification allows for timely genetic counselling and cancer surveillance in both the patient and at-risk family members [5].

Aggressive management, including MMS, was warranted in this case due to several factors: the lesion's facial location, the aggressive histopathological features, and the patient's background of LS. MMS offers precise margin control while preserving healthy tissue, thereby optimizing both oncologic and cosmetic outcomes [2]. Unlike wide local excision (WLE), which samples only a portion of the margins, MMS evaluates 100% of the surgical margins through systematic frozen section analysis, minimizing recurrence risk [2,3]. This approach is particularly recommended for aggressive tumors located in cosmetically sensitive regions [2]. Given the possibility of recurrence and the patient’s syndromic background, aggressive treatment was essential to achieve both oncologic safety and optimal functional and aesthetic outcomes.

To the best of our knowledge, few cases of MTS have been reported among the Indian population in the literature. However, according to the available reports, no instance of MTS associated with SC has been documented in the Indian context so far. Kansal et al. (2014) described a 65-year-old male with sebaceoma on the back and a synchronous colonic adenocarcinoma, fulfilling clinical criteria for MTS, with an extensive family history of visceral malignancies [6]. More recently, Pallikkalakathu et al. (2024) reported a case of synchronous squamous cell carcinoma and colonic adenocarcinoma in a 55-year-old male, with confirmed MSH6 mutation on next-generation sequencing and a positive family history [7]. Notably, while previous Indian reports involved sebaceoma and squamous cell carcinoma, our case involved a histopathologically confirmed SC, a more aggressive neoplasm with higher malignant potential. Additionally, the use of MMS for margin control and tissue preservation in our case underscores the importance of surgical precision in managing syndromic SCs, particularly in cosmetically sensitive sites. This report adds to the growing body of Indian literature on MTS and highlights the diagnostic and therapeutic nuances of managing SC in the setting of LS.

## Conclusions

This report underscores the importance of maintaining a high index of suspicion for MTS in patients with Lynch syndrome presenting with any cutaneous lesions, even at atypical sites. The extraocular location of SC in this patient highlights the need for vigilance beyond the commonly affected periocular region. Histopathology, supported by immunohistochemistry, plays a vital role in confirming the diagnosis and guiding risk stratification. MMS offers superior margin control, particularly in cosmetically sensitive areas. Early dermatologic intervention, coupled with genetic evaluation, plays a pivotal role in reducing morbidity and facilitating familial cancer surveillance.
